# Recurrent Epithelioid Hemangioendothelioma of Calcaneum: A Case Report of a Rare Tumor

**DOI:** 10.7759/cureus.16052

**Published:** 2021-06-30

**Authors:** Ashish Rustagi, Soumyadip Sen, Rajni Prasad, Loveneesh Krishna, Jatin Talwar

**Affiliations:** 1 Orthopaedics, Vardhman Mahavir Medical College and Safdarjung Hospital, New Delhi, IND; 2 Pathology, Vardhman Mahavir Medical College and Safdarjung Hospital, New Delhi, IND

**Keywords:** tumors of calcaneum, bone, recurrent epithelioid hemangioendothelioma, vascular neoplasm, case report

## Abstract

Epithelioid hemangioendothelioma (EHE) is an uncommon malignant vascular tumor characterized by epithelioid or histiocytoid endothelial appearance. Here we present the case of a 65-year-old female with recurrent EHE of the left calcaneum. The patient had developed soft-tissue swelling over the lateral aspect of the left hindfoot three years ago, which was previously managed by excisional biopsy as per medical records and the histopathological examination (HPE) had revealed an EHE. A year later, she again developed a painful swelling with superficial ulceration over the same region and presented to us. A plain radiograph of the foot showed a soft-tissue swelling with cortical breach over the lateral aspect of calcaneum. MRI revealed a mass encasing peroneus tendons, with extension into the lateral surface of calcaneum. CT angiography revealed a mass eroding the lateral cortex of calcaneum and receiving blood supply from calcaneal branches of peroneal artery. PET-CT scan did not reveal any other primary or metastatic site. Core needle biopsy of calcaneum was suggestive of EHE. Limb salvage was difficult as the tumor was recurrent and involved the skin and the weight-bearing part of the calcaneum. The patient was managed with trans-tibial amputation. Immunohistochemical (IHC) staining of the excised tissue was positive for CD34, Vimentin, SMA, and Fli-1. The margins were negative for any tumor cells and she did not require any adjuvant therapy. At two years follow-up, she was free of any further recurrence or metastasis. Recurrent tumors of this variety are fast-growing with metastatic potential and may cause mortality. Hence, they need to be managed aggressively.

## Introduction

Epithelioid hemangioendothelioma (EHE) is a rare tumor of vascular origin, with an epithelioid and histiocytoid appearance. They originate from vascular endothelial or pre-endothelial cells and comprise less than 1% of all vascular tumors [1]. The World Health Organization (WHO) has classified these lesions under the category of locally aggressive tumors with metastatic potential [2]. In our study, we report the case of a 65-year-old female with a recurrent EHE of the left calcaneum with ulceration of the overlying skin.

## Case presentation

A 65-year-old female presented to our outpatient department (OPD) with complaints of recurrent swelling over the lateral aspect of the left foot associated with pain and difficulty in walking for three months. The patient had developed soft-tissue swelling over the lateral aspect of the left foot three years back, which had been treated at an outside facility with an excisional biopsy of the swelling, according to the medical records. The histopathological examination (HPE) had revealed an EHE of the left calcaneum with positivity for Fli-1, CD31, and Vimentin. The patient had not received any adjuvant chemotherapy or radiotherapy after this procedure. A year later, she again developed swelling over the same area with ulceration of the overlying skin, for which she presented to our OPD. The swelling was insidious in onset, gradually progressive in size and associated with pain.

The patient was a known case of type two diabetes mellitus and hypothyroidism for which she was already on medication. She was a non-smoker and non-drinker. She had a normal sleep pattern; there was no history of significant weight loss, and no alteration in appetite and normal bowel and bladder habits. There were no respiratory symptoms or difficulties. There was no positive family history for any cancer.

**Figure 1:**
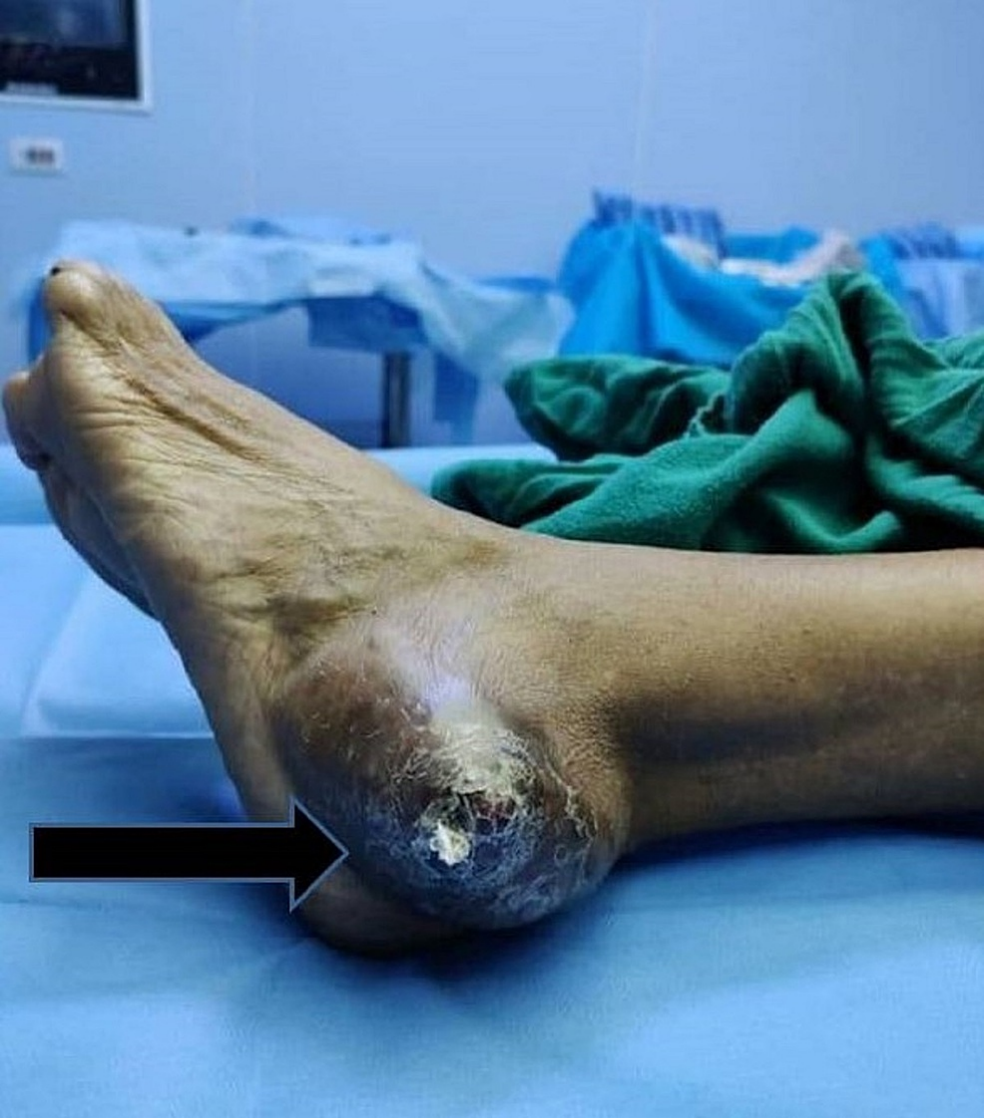
Swelling over the lateral aspect of the left hindfoot

The swelling was 5x4 cm in size, round to oval in shape, located over the lateral aspect of the left hindfoot with superficial ulceration of the overlying skin; no venous prominence, sinus or fistula were present. It was tender to touch, with no superficial rise of temperature. It was pulsatile in nature, immobile in both axes, fixed to the underlying bone and overlying skin. It had well-defined margins and a smooth surface and was compressible but not reducible (Figure 1). There was a single ulcer which was irregular in shape, 0.5 cm in size in the largest dimension in two axes and had an everted edge. There was no discharge and the surrounding skin was pigmented. It was tender on palpation, margin and base were indurated, and it was immobile. There were no palpable lymph nodes.

**Figure 2:**
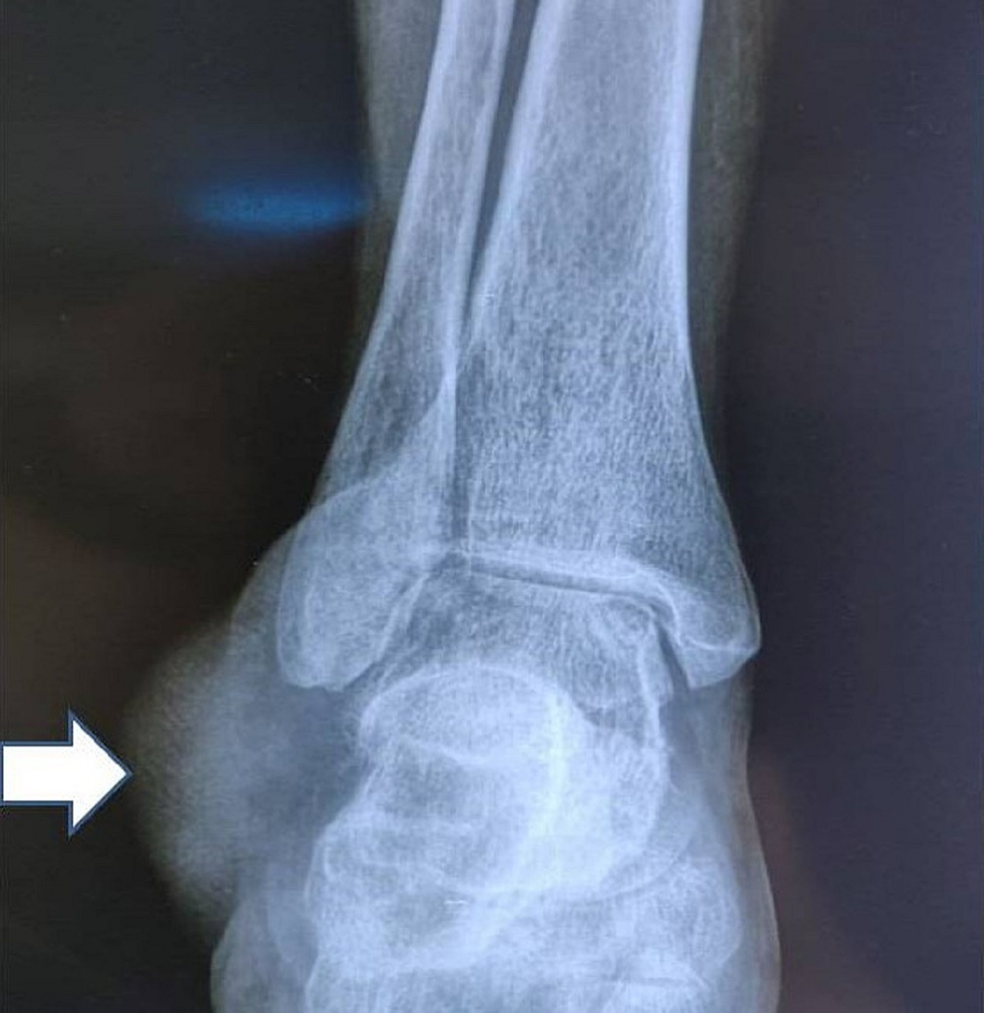
Radiograph showing soft tissue swelling with lucency in the calcaneum

**Figure 3:**
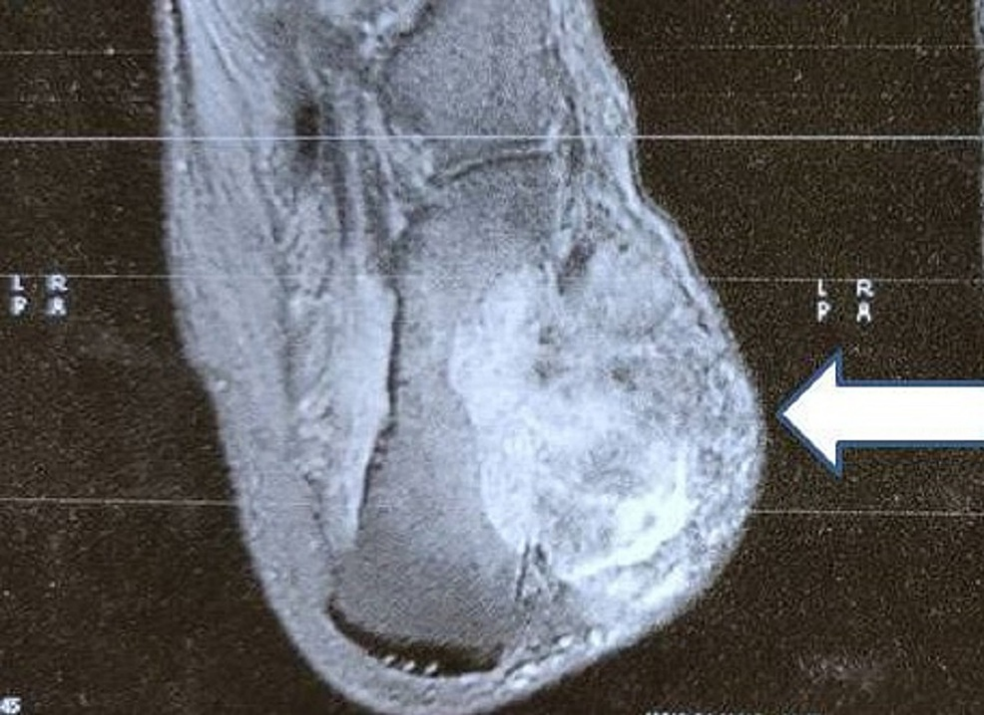
MRI showing the mass involving the calcaneum and subcutaneous tissue MRI: Magnetic Resonance Imaging

**Figure 4:**
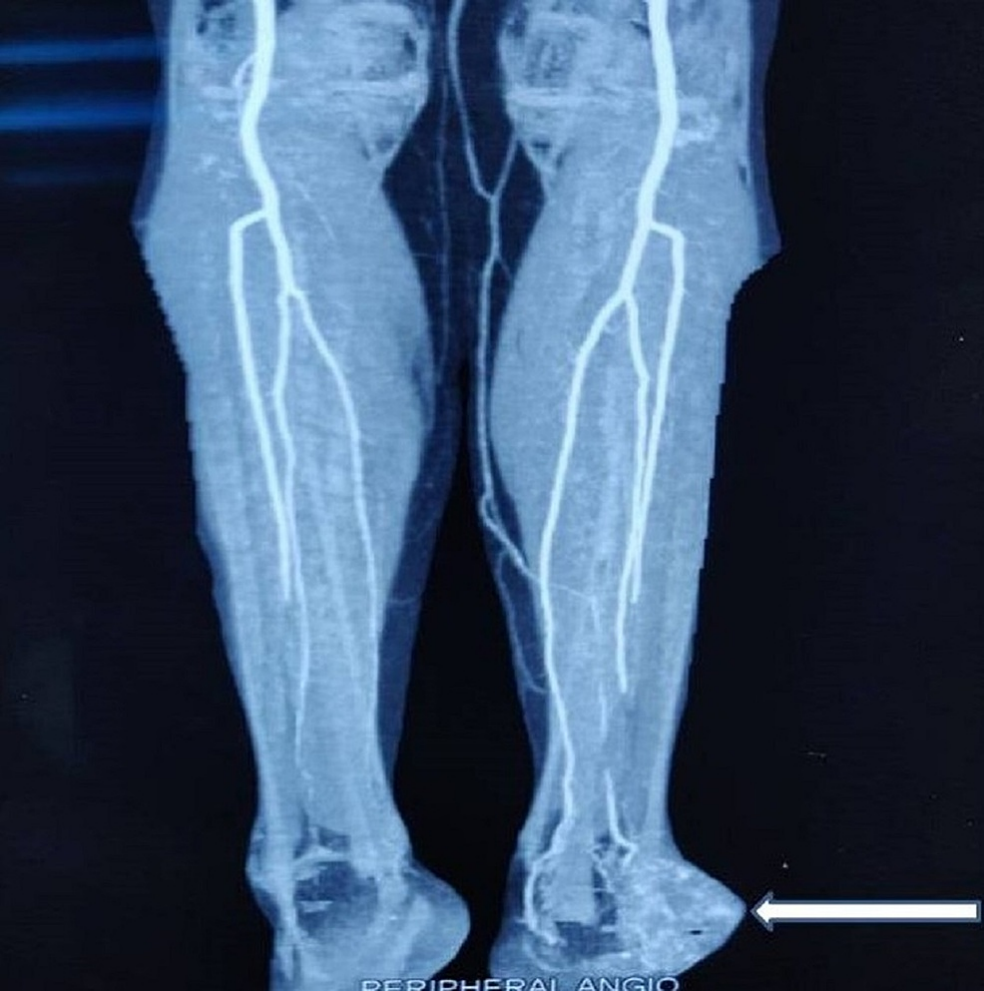
CT angiogram showing the calcaneal branches of peroneal artery feeding the tumor CT: Computed Tomography

The radiographs of the ankle and foot showed a soft-tissue swelling over the lateral aspect of the left foot with lucency in the calcaneum and cortical breach over the lateral aspect of the calcaneum (Figure 2). Magnetic resonance imaging (MRI) of the left ankle joint revealed a well-defined multilobulated mass of 57x40x40 mm size in the lateral ankle and foot, predominantly in the subcutaneous plane, encasing the peroneus tendons. There was intra-osseous extension into the lateral aspect of the calcaneum and extension into muscles inferolateral to the calcaneum (Figure 3). A few dilated perilesional tortuous vessels were also seen. A CT angiogram of the lower limbs was performed which revealed that the tumor was receiving blood supply from the calcaneal branches of the peroneal artery. There was also cortical erosion of the lateral cortex of calcaneum (Figure 4). PET-CT scan there was neither any other site of primary tumor nor any sites of metastasis. We then performed a core needle biopsy of the calcaneum and the HPE was suggestive of an EHE.

We performed a trans-tibial amputation considering the age of the patient, the recurrent nature of the swelling and the chances of metastasis.

**Figure 5:**
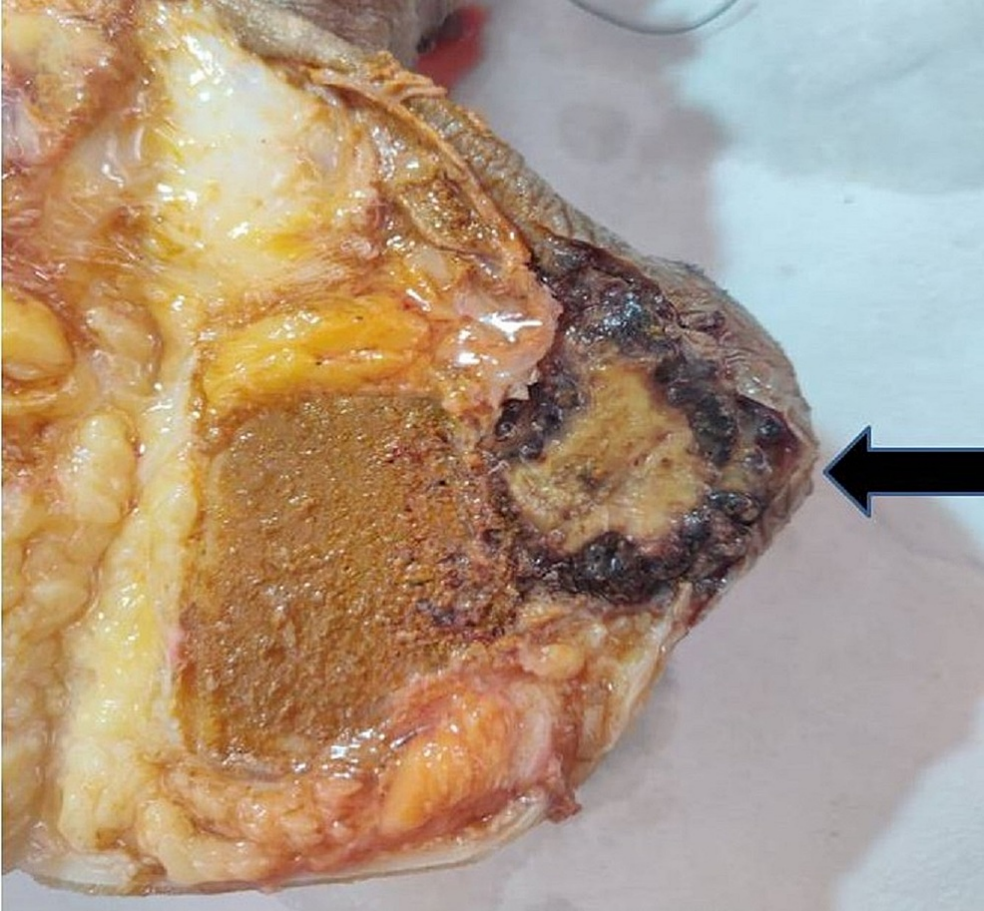
Cut section showing the tumor involving the calcaneum and subcutaneous tissue

**Figure 6:**
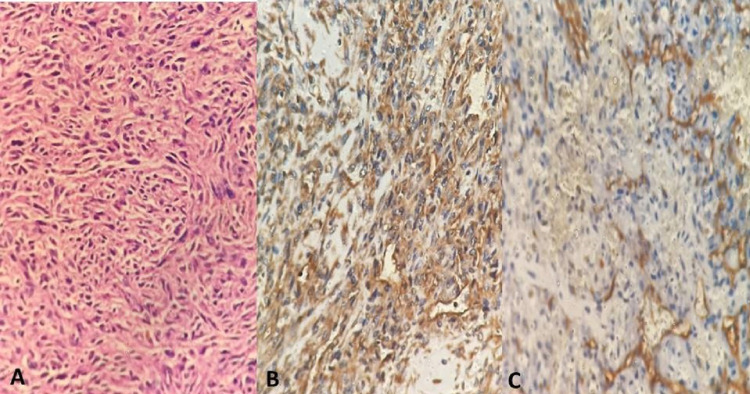
Hematoxylin and eosin stain and IHC staining A: Hematoxylin and Eosin stain showing tumor arising from vessels with mild atypia, nuclear pleomorphism and myxoid changes. B: positive IHC staining with vimentin. C: positive IHC staining with CD34. IHC: Immunohistochemistry

Grossly, the cut section of the hindfoot showed the tumor involving the calcaneum and subcutaneous tissue (Figure 5). HPE of the excised tissue showed a tumor arising from the vessels extending outward from the lumen. The tumor cells showed a mild degree of atypia and nuclear polymorphism, focal spindling and areas of necrosis. Few areas showed mitotic activity and myxoid change. Immunohistochemical (IHC) staining of the excised tissue was positive for CD34, Vimentin, SMA and Fli-1 markers and negative for HMB 45, Desmin, Cytokeratin and S-100 (Figure 6). These markers were confirmatory for the diagnosis of an EHE.

As the margins of the resected tumor were negative for tumor cells, any adjuvant chemo/radiotherapy was not required. The patient was mobilized with a below-knee prosthesis. At two years after surgery, there was no evidence of any recurrence or metastasis.

## Discussion

The term epithelioid hemangioendothelioma (EHE) was first described for a vascular neoplasm of soft tissue by Weiss and Enzinger in 1982 [3]. Deyrup et al. reported 49 cases of EHE originating from soft tissue, where the mean age for this tumor was found to be 49 years, affecting both sexes equally [4]. EHE of the bone can be multi-centric and it appears to have a peak incidence in the second and third decades of life and again in the later decades [5]. EHE of bones is very rare and accounts for 1% of all primary bone neoplasms [6]. The long tubular bones are affected most commonly, followed by the spine [7]. In the lower extremity, the most common locations, in decreasing order for occurrence, are femur, tibia, fibula and the small bones of the feet [8].

Most of the cases of EHE are associated with low mortality but some show metastasis and can result in mortality of a patient [9]. EHE is the most aggressive type of hemangioendothelioma with metastatic potential [3]. Kleer et a.l found that the most important factor in predicting the prognosis in EHE was visceral involvement [5]. The IHC staining for diagnosing EHE can be done using markers like CD31, Fli-1 and CD34. Though CD34 is expressed by more than 90% of vascular tumors, it is also expressed by a variety of soft tissue tumors; hence, it has poor specificity. CD31 is a relatively specific marker for vascular tumors. The endothelium expresses Fli-1 protein; hence, it is useful in identifying vascular neoplasms like EHE [10]. A combination of Fli-1 and CD31 is ideal for the diagnosis [11].

Dechambre et al. reported a case of a 28-year-old woman with pain in the left heel and a lytic polylobulated lesion in the left calcaneus with numerous small nodules in both lungs [12]. The patient was managed with adequate calcaneal resection of lesion. At one year after resection, CT and MRI showed five small asymptomatic hepatic peripheral nodular lesions with no change in the pulmonary lesions [12].

Plumby et al. reported the case of a 60-year-old male who had presented with right foot pain worsened by activities. Initial radiographs were reportedly normal and corticosteroid injection administration into the sinus tarsi did not provide any relief [13]. MRI had revealed a complex lesion in calcaneum with surrounding edema and a CT-guided biopsy revealed a lytic lesion with cortical destruction. IHC stains were positive for CD31, CD34 and CAMTA1. The patient was managed with excision and curettage with argon beam. The lesion was filled with cement and percutaneous pinning was also done. Three years after follow-up there was no recurrence [13].

EHE is associated with a recurrence rate of nearly 10%, in contrast to conventional hemangioma [14]. Broida et al. in 2019 reported a case of a recurrent hemangioendothelioma which was previously managed by radical resection of proximal femur followed by total hip arthroplasty [15]. At eight months of follow-up, an increasing lysis of the ischium of the same side was noted, and MRI and CT angiogram revealed a lobulated expansile mass which was involving the ischium, inferior pubic ramus and posterior acetabular column. The patient was managed with monotherapy of oral propranolol (40 mg twice daily). After four weeks of therapy the pain was stabilized and at five months after therapy, the pain had subsided and the mass had not progressed [15].

Angelini et al. reviewed 62 patients with EHE of bone who were treated from 1985 to 2010 and found that the survival of patients at ten years follow up was 97% for unifocal tumors compared to 74% for multifocal tumors. They also reported a recurrence rate of 25% [16].

Lai et al. reported a case of a two-year-old boy with recurrent and locally metastasizing vascular tumor involving the skin, subcutaneous tissue and muscle of the right forearm, right distal radius and ulna, and multiple lymph nodes of the right axilla [17]. He was ultimately managed with an above elbow amputation of right side with axillary lymph node clearance. Histologically, the tumor had some features of a spindle cell hemangioendothelioma but the low-grade aggressive behavior resembled an EHE [17].

The role of adjuvant chemotherapy and/or radiotherapy is still not clear in cases of EHE. In cases of localized EHE, radiotherapy is usually given after surgical resection to control any residual disease or recurrence. The use of chemotherapy, however, is reserved for cases with widespread disease [10].

The EHE is a very rare tumor of the bone. It may be associated with recurrence and metastasis too, which can alter the prognosis in patients. There is a requirement of prompt diagnosis in these patients and adequate tumor resection too. In our case, we had the option of performing a limb salvage surgery initially with arterial embolization of the vessels feeding the tumor followed by marginal excision of the tumor and reconstruction of the calcaneum with flap coverage of the raw area. Our patient had a rapidly growing recurrent swelling with involvement of the weight-bearing area of the heel and ankle, along with ulceration of the overlying skin, which posed a hindrance to obtaining a wide tumor-free margin. There were risks of flap necrosis and metastasis. Hence, considering the advanced age and co-morbidities like diabetes mellitus and hypothyroidism, duration of the surgery, and need for adjuvant chemo/radiotherapy, we performed a trans-tibial amputation after thorough discussion with the patient and attendants.

## Conclusions

EHE is a rare tumor and recurrence, though rare, is reported in the literature. It is the most aggressive type of hemangioendothelioma with a risk of metastasis, which can result in mortality of the patient. Visceral involvement can worsen the prognosis in such tumors.

Solitary EHE of the calcaneum is extremely rare. Therefore, to effectively manage tumors of this variety, it is imperative to identify them early. Hence, recurrent EHE warrants a quick and thorough workup with prompt and aggressive management.
